# Traumatic contralateral chylothorax in a patient with penetrating neck injury: a case report

**DOI:** 10.1093/jscr/rjab031

**Published:** 2021-03-22

**Authors:** Olumide Olotu, Nneka Uchendu, John Holtrop, Isabella Graham, Joseph Buck

**Affiliations:** St. George’s University, School of Medicine, True Blue, Grenada; Wayne State University, School of Medicine, Detroit, MI, USA; Department of Surgery, Ascension St. John Hospital, Detroit, MI, USA; St. George’s University, School of Medicine, True Blue, Grenada; Department of Surgery, Ascension St. John Hospital, Detroit, MI, USA

## Abstract

Chylothorax is defined as a collection of chyle within the pleural cavity secondary to injury of the thoracic duct. We describe a rare case of a contralateral chylothorax resulting from a penetrating wound to the left lower anterior neck region. A 37-year-old male presented to the emergency room with a penetrating stab wound of the left neck. Upon clinical exam, the wound measured about 3–4 cm with minimal bleeding and no expanding hematoma or other hard sign of vascular injury. Subsequently, his right chest tube output developed a milky appearance with a total volume of 260 cc over 24 h. The specimen was sent for triglyceride analysis and confirmed diagnosis of chylothorax. He was managed with conservative therapy not requiring surgical intervention. The anatomical variations arising in the thoracic duct warrant the consideration of possible chylothorax in both right and left pleural effusions secondary to penetrating trauma.

## INTRODUCTION

Defined as a collection of chyle within the pleural cavity secondary to injury of the thoracic duct, chylothorax is a rare but potential lethal condition. The etiology of chylothorax can either be congenital or acquired and further subdivided into traumatic and nontraumatic chylothorax. Arising from either blunt trauma, penetrating trauma or iatrogenic cases, this condition can be seen in various clinical settings. Nontraumatic etiologies of chylothorax include malignancy, inflammatory response and idiopathic causes [1]. Iatrogenic causes comprise most traumatic etiology of chylothorax while non-iatrogenic causes are exceedingly rare [2]. Here, we describe a rare case of a contralateral chylothorax resulting from a penetrating wound to the left lower anterior neck region.

## CASE REPORT

A 37-year-old male with no significant past medical history presented to the emergency room with a penetrating stab wound to zone one of the left neck. On arrival, the patient had a GCS of 15 and endorsed alcohol intoxication. Upon clinical exam, the wound measured about 3–4 cm with minimal bleeding and no expanding hematoma or other hard sign of vascular injury. Initial blood pressure showed 68/40 mmHg; following IV fluid resuscitation with normal saline and packed red blood cells, repeat blood pressure was 134/85 mmHg. The remaining vital signs were stable. The patient was intubated in the emergency department for airway protection and ATLS protocol was followed. An X-ray of the neck revealed left subcutaneous emphysema. Neck computed tomography angiography was performed to rule out vascular injury. It did reveal a possible right pulmonary laceration at the apex along with a trace right apical pneumothorax. Given the findings on the CTA, the patient was then taken to the OR for esophagography and bronchoscopy which revealed no acute injuries. A right chest tube was placed and monitored for drainage.

On a postoperative day (POD) 1 a repeat chest X-ray demonstrated bibasilar atelectasis; however, he was successfully extubated later that day. On POD 3, the patient became hypertensive and the right chest tube output developed a milky appearance with a total volume of 260 cc over 24 h. The specimen was sent for triglyceride analysis and confirmed diagnosis of chylothorax with a triglyceride level of 1106 mg/dl. Nutrition was consulted and recommended a low-fat diet of 20 g fat/day in addition to MCT Oil. Octreotide was added to his scheduled medications. Chest tube output increased to 340 cc on POD 4 and 700 cc on POD 5 before downtrending to 260 cc on POD 6. The drainage continually decreased in volume until POD 10 when output over 24 h was less 40 cc and the chest tube was successfully removed. On POD 10, the patient was discharged.

## DISCUSSION

Traumatic chylothorax, as seen in our patient, is the most common cause of chylothorax accounting for ~38% of the total number of cases worldwide. Penetrating and blunt trauma accounts for just 5% of traumatic chylothorax and 2% of the total number of chylothorax cases worldwide [3]. Pleural effusions secondary to penetrating chest trauma are much more commonly associated with hemothorax rather than a chyle accumulation. Consideration should be given to an alternative diagnosis such as chylothorax in the setting of milky discharge from the thorax with a triglyceride levels >110 mg/dl and confirmed with the presence of chylomicrons in the fluid sample [3]. Peculiar to our patient was the presence of a right-sided chylothorax. Most chylothorax occurs on the left side due to the native anatomy of the thoracic duct, which typically arises from the cisterna chyli and carries lymph superiorly, crossing to the left side of the spine at the level of T5 [4]. Of note, this typical course of the thoracic duct occurs in only 40–50% of individuals, with several anatomical variations in the course occurring mostly after the aortic hiatus [5]. These variations in the anatomy of the thoracic duct offer a potential explanation as to why our patient who sustained a left-sided penetrating injury ended up with a right-sided chylothorax. The penetrating object extending past the midline to the right side of the thorax lends another explanation to this unusual presentation.

Management of chylothorax begins with draining the lymphatic fluid from the chest with either a thoracocentesis or tube thoracostomy for larger, rapid fluid accumulation [1]. Once the lymphatic fluid has been adequately drained from the chest, clinical focus shifts to controlling the leak. Conservative therapy is typically pursued first to maintain adequate nutrition while minimizing chyle production. In addition to providing nutritional supplementation, several cases of octreotide use in the setting of a chylothorax have been documented. This drug reduces chyle flow by decreasing intestinal production of chyle [6, 7].

In our patient, the chylothorax responded well to conservative measures. However, if conservative therapy fails, surgical intervention may be considered. Conservative therapy is considered a failure if chyle drain output reaches 1000 ml daily for five treatment days, drainage output remains unchanged for >2 weeks or clinical deterioration occurs [8]. The most common surgical procedure for a persistent chylothorax is either a thoracoscopic or an open thoracic duct ligation. Other options include pleuroperitoneal shunt placement and thoracic duct embolization [1].

As seen in this patient, the anatomical variations arising in the thoracic duct warrant the consideration of possible chylothorax in right or left-sided pleural effusions secondary to penetrating trauma.
